# Water and oil-grease barrier properties of PVA/CNF/MBP/AKD composite coating on paper

**DOI:** 10.1038/s41598-023-38941-w

**Published:** 2023-07-29

**Authors:** Shancong Huang, Xiyun Wang, Yu Zhang, Yu Meng, Feiguo Hua, Xinxing Xia

**Affiliations:** 1 College of Textile Science and Engineering (International Institute of Silk), Zhejiang Sci-Tech University, Hangzhou, 310000 Zhejiang China; 2 Zhejiang Jinchang Specialty Paper Co., Ltd., Quzhou, 324404 Zhejiang China

**Keywords:** Materials science, Nanoscience and technology

## Abstract

In this paper, three kinds of micro-nano bamboo powder (MBP) and alkyl ketene dimer (AKD) were added to the polyvinyl alcohol/cellulose nanofiber (PVA/CNF) coating to prepare PVA/CNF/MBP coated paper and PVA/CNF/M-MBP/AKD coated paper. The results showed that MBP improved the oleophobicity of PVA/CNF coating, and the grease resistance grade of PVA/CNF/B-MBP and PVA/CNF/M-MBP coated papers reached the highest level, with a kit number of 12. Among the PVA/CNF/MBP coated papers, the PVA/CNF/M-MBP coated paper has the best hydrophobic properties, with the water contact angle and Cobb value of 74° and 21.3 g/m^2^, respectively. In addition, when the AKD dosage was 0.2% in the PVA/CNF/M-MBP/AKD coating, the kit number of the coated paper was 11, the Cobb value was 15.2 g/m^2^, the water contact angle was 103°, and the tensile strength was found to increase slightly. Therefore, compared with PVA/CNF coated paper, PVA/CNF/M-MBP/AKD coated paper has good strength and excellent hydrophobic and oleophobic properties.

## Introduction

The development of hydrophobic and greaseproof paper has brought great convenience to the field of oil and grease packaging, which involves food, industry, medical and other fields^1^. In order to improve the hydrophobic and oleophobic properties of paper, it is necessary to reprocess the base paper. As we all know, the base paper is composed of plant fibers. The hydrophilicity of cellulose and the porosity of the paper surface determine that the base paper cannot achieve hydrophobic and oleophobic properties^2^. Therefore, it is necessary to apply a coating (spray a dense polymer film or coat an oil-proof agent) on the paper surface to achieve the oleophobicity of the paper.

The polymer is sprayed on the paper surface to form a plastic film, which can effectively prevent the contact between the paper and the grease. However, polymers such as polyethylene, polypropylene, and polyvinyl chloride have stable structures and long natural degradation times, which have an impact on the environment and organisms^3^. The fluorine-containing oil-repellent coating has low surface energy, which results in higher oleophobicity of coated paper. However, fluorine-containing substances are decomposed by high temperature and enters the human body with food, which can cause various chronic diseases and even cancer^4, 5^. Therefore, it is necessary to develop a green and environmentally friendly greaseproof paper to replace the ordinary greaseproof paper.

Tyagi et al.^6^ prepared CNC-composite barrier coatings using cellulose nanocrystals (CNC), nano-filler montmorillonite clay, soy protein and alkyl ketene dimer. It was found that the kit number of CNC-composite coated paper was 6. Similarly, the kit number of cellulose nanofiber/carboxymethyl cellulose coated paperboards prepared by Mazhari Mousavi et al.^7^ was also 6. In addition, Sheng et al.^8^ prepared non-toxic fluoro-free grease-proof papers coated with sodium alginate (SA)/sodium carboxymethyl cellulose and SA/propylene glycol alginate, the maximum of kit number reached 9. The coat weight is one of the important factors affecting the barrier properties of coated paper. Yook et al.^9^ investigated different types of cellulose nanofibrils barrier coatings and their barrier properties and found that a coat weight of at least 10 g/m^2^ was desirable to impart efficient the barrier properties to papers. AKD-CNF coated paper had good hydrophobic properties with water contact angle up to 113°, but its oleophobic properties was still not good with kit number of 9.

Polyvinyl alcohol (PVA) and cellulose nanofibers (CNFs) are suitable coating materials with good film-forming properties and are biodegradable^10, 11^. Fadel et al.^12^ studied CNFs were isolate from sugar beet pulp (SBP) using two methods, they found that ADCNF films had a better performance than DHCNF films with water contact angle of 59.0° ± 1.73, and oil penetrates paper for more than 45 min (coating volume 10 g/m^2^). In addition, they also studied CNF/chitosan nanoparticles (CHNP) coated paper and found that the water sorption 81 g/m^2^ and oil resistance was 78 s^13^. According to the author's previous research, it was found that PVA/CNF coating have hydrophobic and oleophobic properties, but the hydrophilicity of PVA and the lipophilicity of CNFs limit the further improvement of the hydrophobicity and oleophobicity of PVA/CNF coating^14^. Therefore, it is a challenge to improve the hydrophobicity and oleophobicity of PVA/CNF coated paper.

For sustainable development, some natural materials are used to make oil-repellent coatings, such as starch, chitosan, montmorillonite, etc.^15^. However, starch and chitosan were used to prepare grease resistant paper, but the process was complicated and less viable for real-world applications^16^. Park et al.^17^ found that isolated soy protein (ISP) was used as a plasticizer with good oil resistance. ISP-coated paper was resistant to 2 h of grease, and its performance is better than that of ordinary polyethylene composite food packaging paper. Sirvio et al.^18^ reported a cellulose/alginate composite film, which was synthesized by ionic cross-linking and was able to resist turpentine oil for 30 min without permeation. There are also some researchers who directly modified cellulose to obtain oil-repellent cellulose-based materials^19, 20^. However, these preparation processes are complex, require additional chemical raw materials, and are difficult to apply in practice.

Bamboo powder (BP) is widely used in the manufacture of paper products because of its low price, easy availability of raw materials, and chemical composition like wood^21, 22^. BP improves the oleophobic properties of paper due to the presence of lignin^23^. Compared with BP, micro-nano bamboo powder (MBP) not only retains a large amount of lignin, but also has a larger specific surface area, which is more suitable for the preparation of oleophobic coating materials^24^. Alkyl ketene dimer (AKD) is a commonly used hydrophobic agent in the papermaking process. Furthermore, it has low surface energy and good film-forming properties. Hence, the addition of MBP and AKD to PVA/CNF coating can further improve the hydrophobicity and oleophobicity of the coated paper.

In this study, we prepared and characterized three kinds of MBP. Based on PVA/CNF coating, MBP and AKD were added to prepare PVA/CNF/MBP coating and PVA/CNF/M-MBP/AKD coating. In addition, the effects of MBP and AKD on the properties of coated paper were studied, which provided a method for the development of natural degradable and environmentally friendly hydrophobic and oleophobic coating.

## Rationale

**Figure 1:**
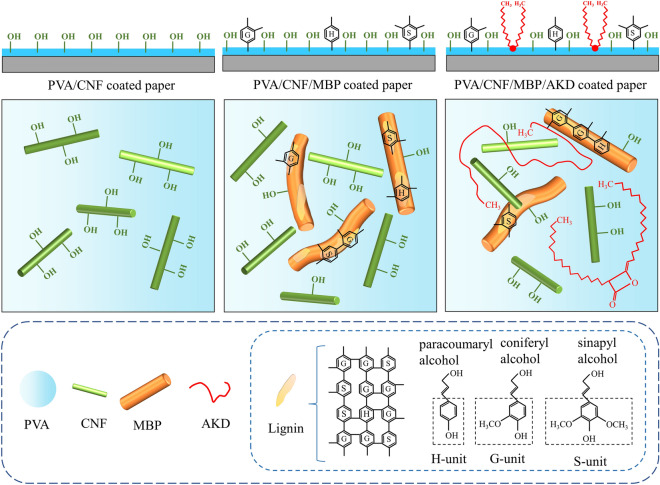
Schematic of hydrophobic and oleophobic based on PVA/CNF coated paper.

The theme underlying the current work is illustrated in Fig. 1. Generally, the PVA/CNF coating have film-forming properties, which impart good barrier properties to paper^25, 26^. However, the surface of PVA and CNFs contains a large number of hydroxyl groups, which cannot avoid the penetration of water and oil. In this paper, the PVA/CNF/MBP coatings were prepared by adding MBP to PVA/CNF coating. We found that the oleophobic properties of the coated paper was significantly improved, and the surface of the MBP contained lignin^27^. Lignin redistribution on paper surface showed excellent grease barrier^28^. The exact reason is unknown but one hypothesis is that lignin has a lower surface energy compared to cellulose^29^. However, the hydrophobic properties of PVA/CNF/MBP coated paper decreased, which was attributed to the hydrophilic cellulose hydroxyl groups in MBP^30^. Finally, AKD was selected as the hydrophobic agent, because the long-chain alkyl groups (hydrophobic groups) of AKD are distributed on the surface of the coating liquid, which can effectively prevent the penetration of water^6, 31^. On the other hand, the long-chain alkyl groups of AKD are lipophilic, so the oil repellency of AKD is weak^32^. Overall, MBP and AKD result PVA/CNF coating with excellent oleophobic and hydrophobic properties. The hydrophobic and oleophobic schematic of PVA/CNF/MBP/AKD coated paper is shown in Fig. 1.

## Materials and methods

### Materials

Bamboo powder (125 mesh, chemical composition: 49.6 wt% cellulose, 16.3 wt% hemicellulose, 24.2 wt% lignin and 9.9 wt% others), cellulose nanofibers ([C_6_H_10_O_5_]_n_) and cellulase (enzyme activity of 16,000 HCU/g) were obtained from Zhejiang Jinjiahao Green Nanomaterials Co., Ltd. (Quzhou, China). The base paper (Table 1) was obtained from Zhejiang Jinchang Specialty Paper Co., Ltd. (Quzhou, China). Alkyl ketene dimer, citric acid (C_6_H_8_O_7_, analytical reagent (AR) grade), sodium citrate (C_6_H_5_Na_3_O_7_, AR grade), sodium hydroxide (NaOH, AR grade), polyvinyl alcohol ([C_2_H_4_O]_n_, AR grade), and other chemical reagents were purchased from Sinopharm Chemical Reagent Co., Ltd. (Shanghai, China).

**Table 1:** Physical properties of base paper.

Basis weight (g/m^2^)	Thickness (mm)	Apparent density (g/cm^3^)	Tensile indices (N m/g)		Kit number	Cobb (g/m^2^)	Contact angles (°)
			MD	CD			
110	0.126	0.87	60.73	34.74	0	17.8	95

The base paper refers to abrasive base paper.

### Preparation

#### Preparation of citric acid-sodium citrate solution

The citric acid and sodium citrate were respectively prepared to a concentration of 0.1 mol/L, then their ratios were adjusted, and the pH value of the mixed solution was measured using a pH meter (PHS-3E, Shanghai INESA Scientific Instrument Co., Ltd., Shanghai, China). Finally, a citric acid-sodium citrate solution with pH 5 was prepared.

#### Preparation of mechanical micro-nano bamboo powder

The BP and water were mixed to prepare a 1 wt% suspension, and then ground with a colloid mill (JM-L65, Wenzhou Longwan Yongxing Huawei Machinery Factory, Zhejiang, China) for 30 min to obtain mechanical pretreatment bamboo powder (M-PBP). Then, the M-PBP was homogenized 5 times in a high-pressure homogenizer (AH-pilot2018, Shanghai Dibosi Biological Technology Co., Ltd., Shanghai, China) at a pressure of 1000 bar to obtain mechanical micro-nano bamboo powder (M-MBP).

#### Preparation of biological micro-nano bamboo powder

10 g BP and 60 U/g cellulase were added to 250 ml of citric acid-sodium citrate solution and pretreated at 50 °C for 6 h. Then the temperature was raised to 90 °C for 30 min to stop the enzymatic reaction. BP was washed to neutrality using a centrifuge (80-2, Changzhou Langbo Instrument Manufacturing Co., Ltd., Jiangsu, Zhejiang) to obtain biological pretreatment bamboo powder (B-PBP). Finally, the B-PBP was homogenized 5 times under the pressure of 1000 bar to obtain the biological micro-nano bamboo powder (B-MBP).

#### Preparation of chemical micro-nano bamboo powder

BP (10 g) and NaOH solution (500 ml, 1 wt%) were mixed in a beaker and stirred at 90 °C (500 r/min) for 1 h. Then, the BP was washed to neutrality using a centrifuge to obtain chemical pretreatment bamboo powder (C-PBP). Finally, the C-PBP was homogenized 5 times under the pressure of 1000 bar to obtain chemical micro-nano bamboo powder (C-MBP).

#### Preparation of coated paper

**Figure 2:**
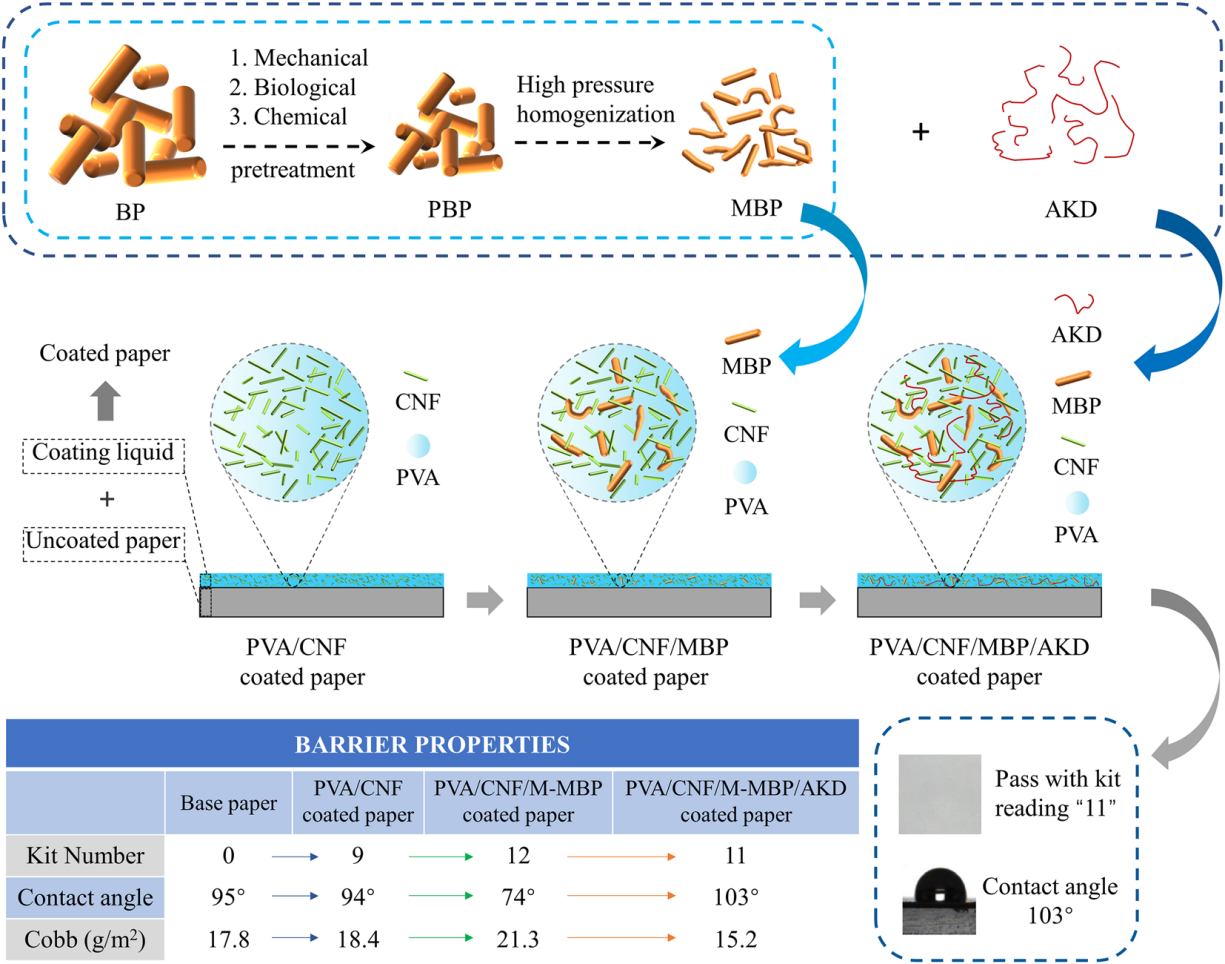
The preparation process and properties of PVA/CNF/M-MBP/AKD coated paper.

PVA and CNFs (3 wt%) were fully dissolved and mixed in 90 °C water, and dispersed in an ultrasonic cleaner (KQ-30DE, Kunshan Ultrasonic Instrument Co., Ltd., Jiangsu, China) for 15 min to obtain CNF/PVA coating. Then, MBP and AKD were added to the PVA/CNF coating for uniform dispersion to obtain PVA/CNF/MBP and PVA/CNF/M-MBP/AKD coatings. The prepared coatings were coated on the surface of the base paper with a hand coater (ZAA 2300, Zehntner, Switzerland), and then dried using electric thermostatic drying oven (DHG-907A 9140A, Shanghai Yiheng Scientific Instrument Co., LTD, Shanghai, China) at 80 °C to obtain PVA/CNF, PVA/CNF/MBP and PVA/CNF/M-MBP/AKD coated paper with a coating weight of 2.0 g/m^2^. The coating thickness was achieved by changing the diameter of the Meyer rod. Coat weight was calculated by measuring the dried weights of the papers before and after coating. The preparation process and properties of PVA/CNF/M-MBP/AKD coated paper are shown in Fig. 2.

### Characterization

The crystal structure of BP and MBP were analyzed by X-ray diffractometer (ARL XTRA, Thermo ARL, Switzerland). The samples were scanned at 40 kV and 30 mA in a 2 h range between 10° and 40° using Cu Kα radiation (λ = 15.4 × 10^–2^ nm) at 1°/min^19^. The crystallinity index (CrI) was calculated according to the empirical method developed by Segal et al.^33^ using the Eq. (1).

1 $$CrI\left(\%\right)=\frac{{I}_{200}-{I}_{am}}{{I}_{200}}$$

where I_200_ is the maximum intensity of the (200) plane (I_200_, 2θ = 22°) that represents the crystalline and amorphous material, and I_am_ is the minimum intensity between plane (110) and (200) (I_am_, 2θ = 18°) that represents the amorphous material. The chemical structures of BP and MBP were analyzed by infrared spectrometer (Nicolet 5700, Thermoelectric Corporation of America). The morphology of BP and MBP were observed with a desktop scanning electron microscope (Phenom pro, Funer Scientific Instruments (Shanghai) Co., Ltd.). The surface morphology of CNFs was observed by atomic force microscope (XE-100E, PSIA, Korea). The scanning electron microscope (SEM) images of BP and MBP and the atomic force microscope (AFM) images of CNFs were imported into Nanoscope analysis and Nano measurer software, and 100 CNFs and BPs were randomly selected to measure their size. In addition, the obtained data were graphed and analyzed using Origin software.

Before test, the papers were placed at 23 ± 2 °C under a relative humidity of 50 ± 2% for 24 h. The oil/grease resistance of paper was measured using the TAPPI T559 standard. The water contact angle of paper was measured according to ASTM D 724-1999 standard, using a surface contact angle tester (OCA40, Dataphysics, Germany). Water absorption of paper (Cobb test) was determined using Paper Cobb Water Absorption Tester (PWA-01, Sichuan Changjiang Paper Instrument Co., Ltd., China) according to TAPPI T441 standard. The tensile strength of paper was determined using a computerized tensile tester (TTM, Hangzhou Qingtong Boke Automation Technology Co., Ltd., China) according to the TAPPI T494 standard.

## Results and discussion

### Characterization of MBP

#### X-ray diffraction analysis

**Figure 3:**
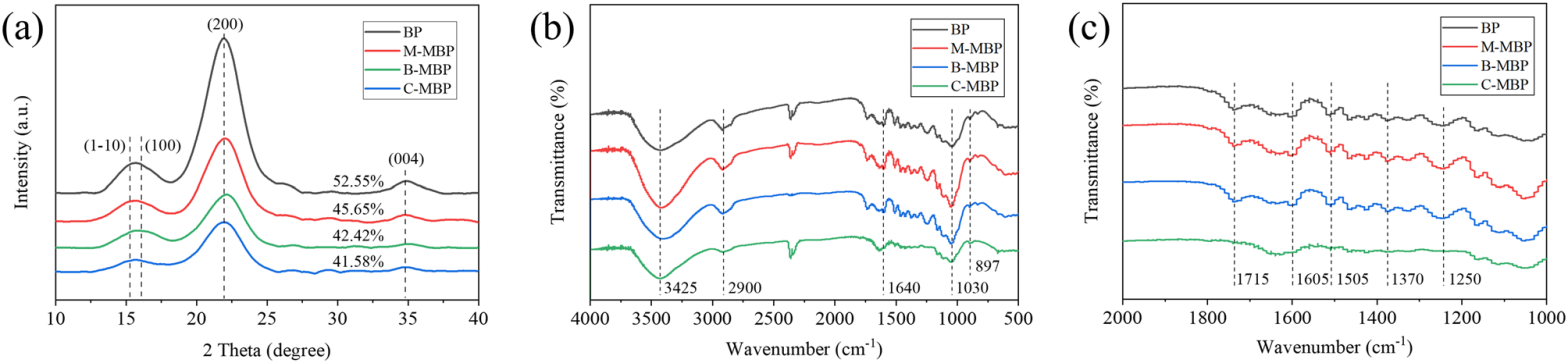
(**a**) XRD patterns of BP, M-MBP, B-MBP and C-MBP, (**b**) and (**c**) FTIR spectra of BP, M-MBP, B-MBP and C-MBP.

The crystalline properties of BP, M-MBP, B-MBP and C-MBP were performed according to X-ray diffraction (XRD). As is shown in Fig. 3a, there was no significant difference in the diffraction peaks of the four bamboo powders. The diffraction overlapped peak at about 16° correspond to the (1-10)/(110) cellulose crystallographic plane, and the diffraction peaks of 22° and 34.5° correspond to the (200) and (004) cellulose crystallographic plane, respectively, which belong to the typical cellulose type I crystal^34, 35^. The CrI of BP was 52.55%, which was higher than the other three MBPs. After high-pressure homogenization of BP, the crystalline region in BP was damaged by mechanical shearing, so that the CrI of MBP was lower than that of BP^36^. However, the CrI of M-MBP was greater than that of B-MBP and C-MBP, which was due to the mechanical pretreatment reduced the size of the BP and did not change the internal structure of the BP. The existence of lignin on the BP surface reduces the shearing effect of the high-pressure homogenization process on the BP and protects the crystallization area of the BP, therefore the crystallinity of M-MBP decreases less. However, both biological and chemical pretreatments changed the internal structure of BP and reduced the lignin content, hence the crystallinity of B-MBP and C-MBP decreased significantly^37^.

#### FTIR spectroscopy analysis

Figure 3b shows the Fourier transform infrared (FTIR) spectra of BP and MBP. It could be seen from the figure that the characteristic peaks of M-MBP and B-MBP are like BP. The characteristic peaks of cellulose appeared in these three infrared spectra, which were the stretching vibration peak of –OH at 3425 cm^−1^, the stretching vibration peak of –CH at 2900 cm^−1^ and the absorption peak of –CO at 1030 cm^−1^, and the hemicellulose absorption peak of –CO at 1640 cm^−1^ (Fig. 3b)^38^. Meanwhile, there were absorption peaks at 1715 cm^−1^, 1605 cm^−1^, 1505 cm^−1^, 1370 cm^−1^, and 1250 cm^−1^ representing lignin on the three infrared spectra, which can prove that BP contains a large amount of lignin (Fig. 3c)^39^. There were also characteristic peaks of cellulose and hemicellulose in the infrared spectrum of C-MBP, but the characteristic peaks of lignin were weak, which indicated that part of the lignin was removed after the BP was pretreated with NaOH solution.

#### Morphology analysis

**Figure 4:**
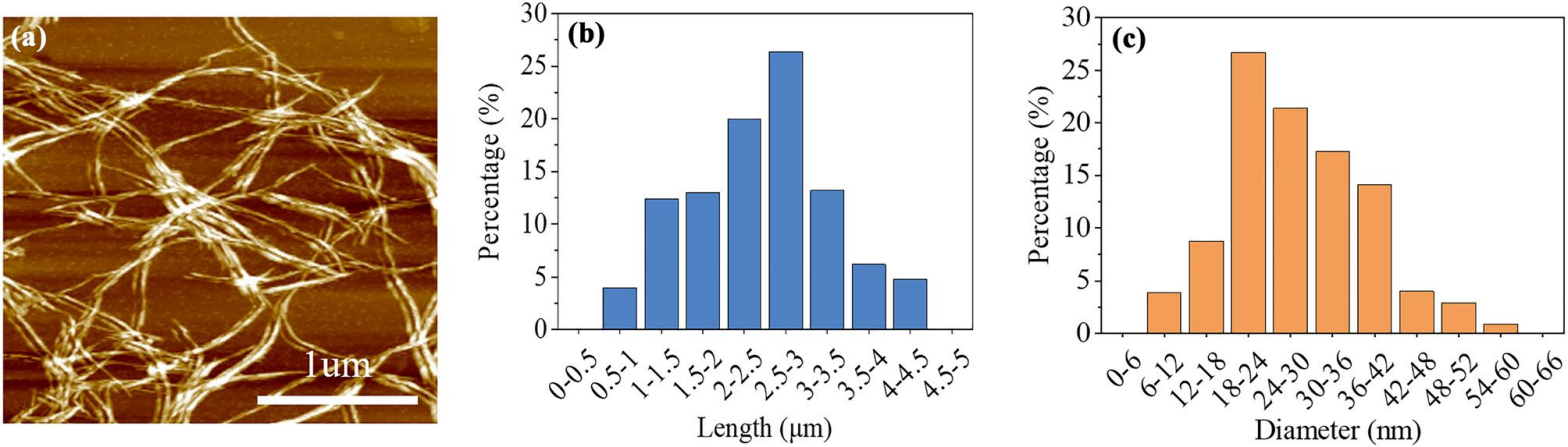
Atomic force microscope images of (**a**) CNFs; (**b**) CNF length and (**c**) CNF diameter.

Figure 4a shows the image of CNF tested with the atomic force microscope (AFM). As shown in Fig. 4a, the CNF was a filamentous structure. And the length and diameter of CNFs were mostly distributed between 1–3.5 µm and 18–42 nm, with an average length of about 2.4 µm and an average diameter of about 28.7 nm (Fig. 4b and c).

**Figure 5:**
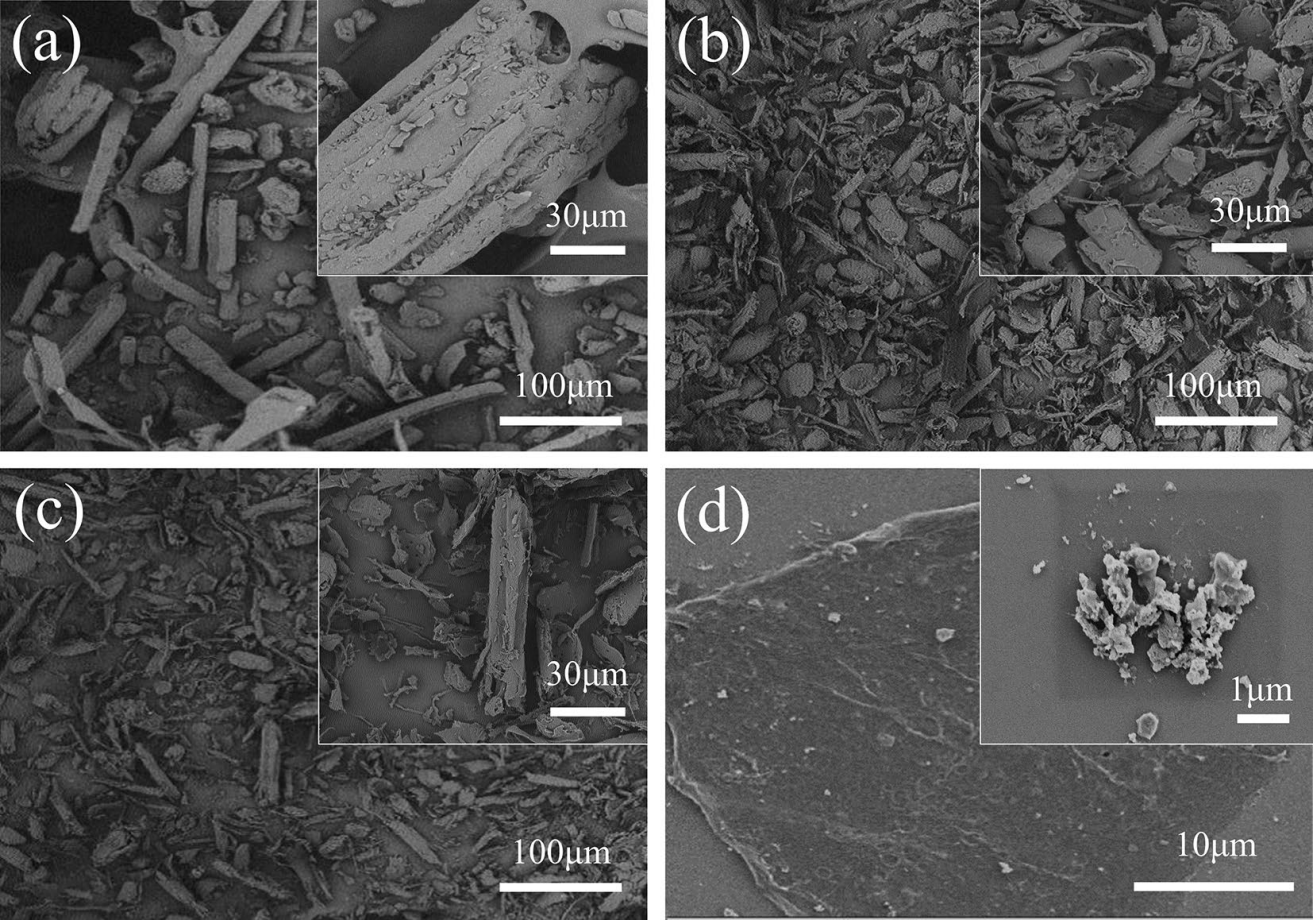
SEM images of (**a**) BPs, (**b**) M-MBP, (**c**) B-MBP and (**d**) C-MBP.

Figure 5 and Table 2 shows the SEM images and sizes of BP and MBP. Figure 5a shows the SEM image of BP, which shows that most of the BP has a rod-like structure with an average length of 166.8 µm, an average width of 23.5 µm, and an average thickness of 782 nm. The M-MBP and B-MBP were mainly sheet-like structures, with a small amount of rod-like structures (Fig. 5b,c). Figure 5d shows that the C-MBP was a staggered and overlapping sheet-like structure with an average length, width and thickness of 758.2 nm, 487.5 nm and 70.3 nm, respectively (Table 2). The size of C-MBP in the MBPs was the smallest. Therefore, the chemical pretreatment has the greatest damage to the structure of the BP and the removal of the lignin from the BP the most. In short, the tiny size of MBP is more beneficial to the film formation of coatings than the BP.

**Table 2:** Physical properties of BP, M-MBP, B-MBP and C-MBP.

Type	Average length (µm)	Average width (µm)	Average thickness (nm)
BP	166.8	23.5	782
M-MBP	43.8	11.6	410
B-MBP	30.8	12.2	390
C-MBP	0.758	0.488	70.3

### Properties of PVA/CNF/MBP coated paper

#### Grease resistance

**Figure 6:**
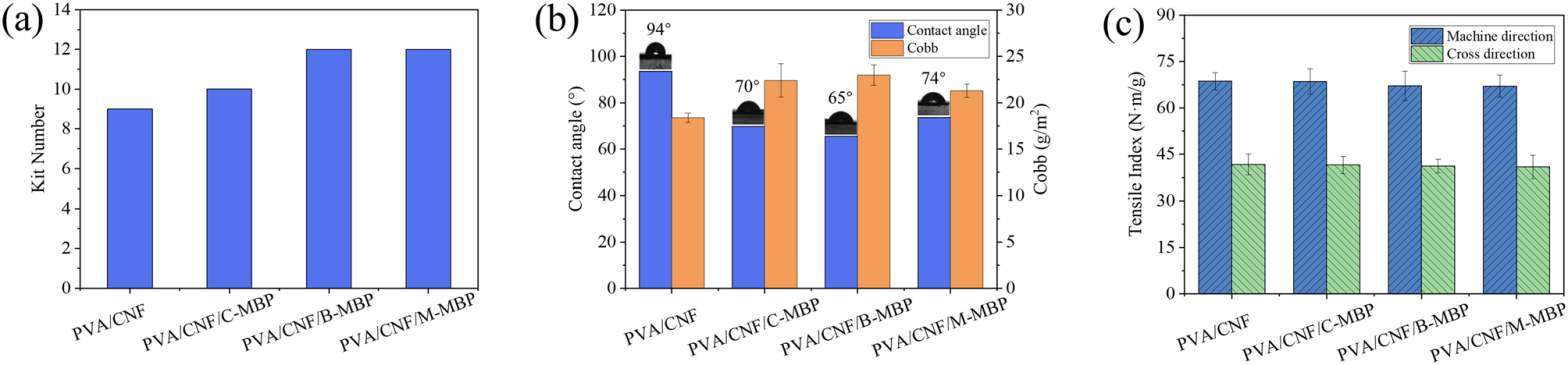
(**a**) Effect of MBP on the grease resistance of paper, (**b**) effect of MBP on hydrophobic properties of paper and (**c**) effect of MBP on tensile strength of paper.

The effect of MBP on the grease resistance of paper is shown in Fig. 6a. It can be seen from the figure that the grease resistance grade of PVA/CNF coated paper was 9, the PVA/CNF/C-MBP coated paper was 10, and the PVA/CNF/B-MBP and PVA/CNF/M-MBP coated paper was 12. Obviously, the MBP improved the grease resistance performance of PVA/CNF coating. Lignin exists on the surface of MBP, and its polarity and surface energy are low, which improves the surface tension of the coatings^23^. This is the fundamental reason why MBP improves the grease resistance performance of coatings. The C-MBP pretreated with NaOH had less grease resistance performance than B-MBP and M-MBP due to the removal of more lignin.

#### Water contact angle and Cobb value

The effect of MBP on the hydrophobic properties of paper is shown in Fig. 6b. The hydrophobicity of PVA/CNF coated paper was the best, with a water contact angle of 94° and a Cobb value of 18.4 g/m^2^. Conversely, the hydrophobic properties of PVA/CNF/MBP coated paper were all worse than PVA/CNF coated paper. Among the samples, the PVA/CNF/B-MBP coated paper had the worst hydrophobicity, with water contact angle and cobb value of 65° and 23.0 g/m^2^, respectively. The MBP added in the coating cannot form an effective combination with PVA, which destroys the compactness of the coating, thus reducing the hydrophobicity of the coated paper^40^. In addition, the lignin in MBP contains hydroxyl groups, and the hydrophilicity of hydroxyl groups is one of the reasons why the hydrophobicity of coated paper decreases^41^.

#### Strength properties

The machine direction (MD) and cross direction (CD) tensile indices of the PVA/CNF coated paper were 68.1 and 41.7 N m/g, respectively. However, the tensile strengths of PVA/CNF/M-MBP coated paper was the smallest, which were 67.17 and 40.92 N m/g (Fig. 6c). Therefore, MBP has a weak effect on the tensile strength of paper. On the other hand, the tensile strength of PVA/CNF/MBP coated paper decreased slightly compared with PVA/CNF coated paper. Cellulose was covered by lignin on the surface of MBP, which affected the binding force of CNFs and PVA^42^. Thus, the tensile strength of PVA/CNF/MBP coated paper decreases.

### Properties of PVA/CNF/M-MBP/AKD coated paper

Compared with the PVA/CNF coating, the PVA/CNF/MBP coating improved the oleophobic properties of the paper and basically maintained the physical strength of the paper, but the hydrophobicity of the paper decreased. AKD is used as a hydrophobic agent in the papermaking process because of its low surface energy and good film-forming properties. In the above studies, the preparation method of M-MBP is the simplest, and the hydrophobic property of PVA/CNF/M-MBP coated paper is the best. Therefore, AKD (relative to PVA dosage) was added to the PVA/CNF/M-MBP coating to form a PVA/CNF/M-MBP/AKD coating, and the hydrophobic and oleophobic properties of the coated paper were further studied.

#### Grease resistance

**Figure 7:**
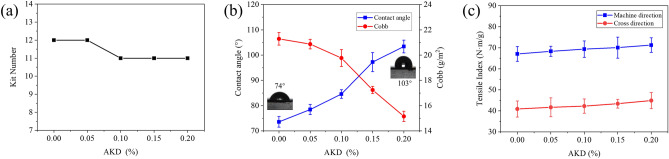
(**a**) Effect of AKD dosage on the grease resistance of paper, (**b**) effect of AKD dosage on hydrophobic properties of paper and (**c**) effect of AKD dosage on tensile strength of paper.

The effect of AKD on the grease resistance of paper is shown in Fig. 7a. The AKD dosage was less than 0.05%, which did not change the oleophobic properties of the coated paper. Meanwhile, the kit number of the coated paper was 12. When the AKD dosage exceeded 0.05%, the oleophobic properties of the coated paper decreased. In addition, the AKD dosage was between 0.10 and 0.20%, and the kit number of the coated paper was 11. AKD is a cationically charged dispersion, which was believed to adsorb primarily ionically to anionically charged lignin^43^. The long-chain alkyl group of AKD is a lipophilic group. Therefore, when the oil/grease contacts the surface of the coated paper, the alkyl group absorbs the oil/grease and penetrates the inside of the paper, and the oleophobic properties of the coated paper decreases^6^.

#### Water contact angle and Cobb value

As the increase of AKD dosage, the hydrophobic properties of the coated paper were significantly improved. Moreover, with an increase of AKD dosage, the Cobb value of the coated paper decreased and the water contact angle increased, as shown in Fig. 7b. When the AKD dosage was 0.2%, the Cobb value and contact angle of the PVA/CNF/M-MBP/AKD coated paper were 15.2 g/m^2^ and 103°. Compared with PVA/CNF/M-MBP coated paper, the Cobb value of PVA/CNF/M-MBP/AKD coated paper decreased by 28.80%, and the water contact angle increased by 40.59%. The reason for this phenomenon is that the AKD molecule contains hydrophobic groups and reactive groups. The reactive group reacts with the carbonyl group of the fiber to form a covalent bond and form a stable film on the fiber surface. On the other hand, the hydrophobic groups (long-chain alkyl groups) are turned away from the fiber surface, so the coated paper has better hydrophobic properties^44^.

#### Strength properties

The effect of AKD dosage on the tensile strength of paper is shown in Fig. 7c. The tensile strength of the coated paper increased slightly with increasing AKD dosage. The MD and CD tensile indices of the non-AKD coated paper were 67.08 N m/g and 40.92 N m/g, respectively. When the AKD dosage increased to 0.2%, the MD and CD tensile indices of the coated paper increased to 71.28 N m/g and 44.92 N m/g, the MD and CD tensile index increased by 6.3% and 9.78%, respectively. The improvement of the tensile strength of the paper is due to the cross-linking of AKD and fibers at high temperature, and the esterification reaction of AKD and the carbonyl group of the fiber to form a covalent bond^45^. Therefore, the addition of AKD is beneficial to improve the tensile strength of the coated paper.

#### Morphology analysis

**Figure 8:**
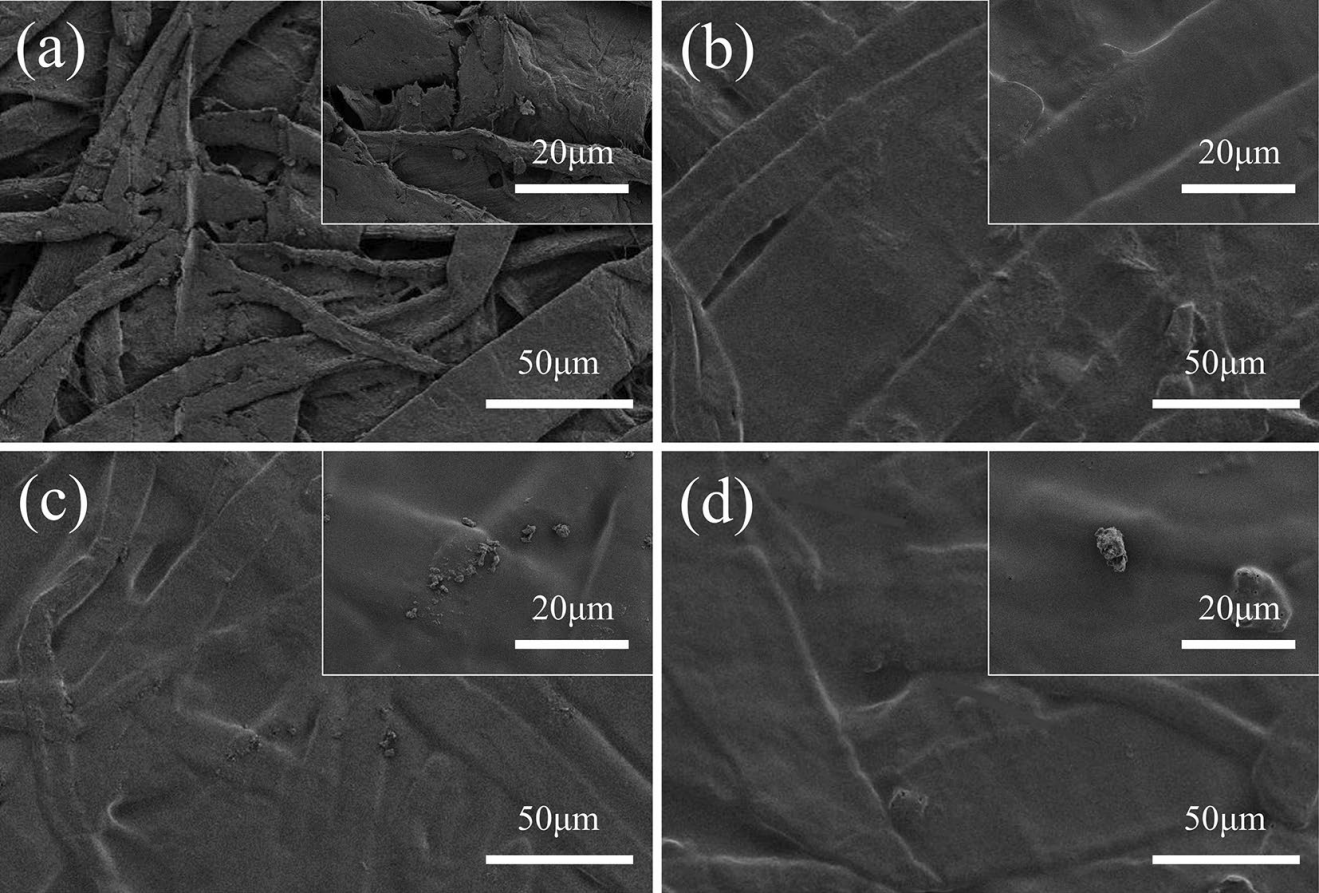
SEM images of (**a**) base paper, (**b**) PVA/CNF coated paper, (**c**) PVA/CNF/M-MBP coated paper and (**d**) PVA/CNF/M-MBP/AKD coated paper.

Figure 8 shows the SEM images of base paper and coated paper. Compared to the coated paper, there are a lot of gaps between the plant fibers in the base paper (Fig. 8a), which allow the liquid to penetrate the interior of the paper through capillary action. This is the reason that the base paper does not have hydrophobic and oleophobic properties. The excellent film-forming properties of PVA and CNF delivers PVA/CNF coated paper the hydrophobic and oleophobic effects by filling the pores between fibers (Fig. 8b). As shown in Fig. 8c and d, the surface of the coated paper was covered by a coating film with M-MBP particles. The low surface energy of M-MBP and the hydrophobic group of AKD would be helpful for increasing the hydrophobic and oleophobic properties of PVA/CNF coated paper.

## Conclusion

In this work, we developed a facile strategy to improve the hydrophobicity and oleophobicity of PVA/CNF coated paper. Analysis of PVA/CNF and PVA/CNF/MBP coated paper with a coating weight of 2.0 g/m^2^, the results showed that the grease resistance grade of PVA/CNF, PVA/CNF/C-MBP, PVA/CNF/B-MBP and PVA/CNF/M-MBP coated papers were 9, 10, 12 and 12. Obviously, MBP improves the oleophobicity of PVA/CNF coating. However, the hydrophobicity and strength properties of the PVA/CNF/MBP coated paper were slightly decreased compared to the PVA/CNF coated paper. Therefore, it is necessary to use AKD to improve the properties of PVA/CNF/MBP coated paper. When the AKD dosage was 0.20%, the Cobb value of PVA/CNF/M-MBP/AKD coated paper decreased by 28.80%, the water contact angle increased by 40.59% and the MD and CD tensile index increased by 6.3% and 9.78%, compared with PVA/CNF/M-MBP coated paper. Overall, compared with PVA/CNF coated paper, PVA/CNF/M-MBP/AKD coated paper has good strength and excellent hydrophobicity and oleophobicity. This is of great significance for the development of environmentally friendly hydrophobic and oleophobic packaging materials.

## Data Availability

All data generated or analysed during this study are included in this published article.

## Abbreviations

PVA: Polyvinyl alcohol
CNF: Cellulose nanofibers
BP: Bamboo powder
MBP: Micro-nano bamboo powder
M-MBP: Mechanical micro-nano bamboo powder
B-MBP: Biological micro-nano bamboo powder
C-MBP: Chemical micro-nano bamboo powder
AKD: Alkyl ketene dimer
ISP: Isolated soy protein
XRD: X-ray diffraction
FTIR: Fourier transform infrared
MD: Machine direction
CD: Cross direction

## References

[1] Hubbe MA, Pruszynski P (2020). Greaseproof paper products: A review emphasizing ecofriendly approaches. BioResources.

[2] Kjellgren H, Engström G (2005). The relationship between energy requirement and barrier properties in the production of greaseproof paper. Tappi J..

[3] Miao Y, von Jouanne A, Yokochi A (2021). Current technologies in depolymerization process and the road ahead. Polymers.

[4] Tokranov AK (2018). How do we measure poly-and perfluoroalkyl substances (PFASs) at the surface of consumer products?. Environ. Sci. Technol. Lett..

[5] Schaider LA (2017). Fluorinated compounds in US fast food packaging. Environ. Sci. Technol. Lett..

[6] Tyagi P, Hubbe MA, Lucia L, Pal L (2018). High performance nanocellulose-based composite coatings for oil and grease resistance. Cellulose.

[7] Mazhari Mousavi S, Afra E, Tajvidi M, Bousfield D, Dehghani-Firouzabadi M (2017). Cellulose nanofiber/carboxymethyl cellulose blends as an efficient coating to improve the structure and barrier properties of paperboard. Cellulose.

[8] Sheng J, Li J, Zhao L (2019). Fabrication of grease resistant paper with non-fluorinated chemicals for food packaging. Cellulose.

[9] Yook S (2020). Barrier coatings with various types of cellulose nanofibrils and their barrier properties. Cellulose.

[10] Wang JW (2018). Moisture and oxygen barrier properties of cellulose nanomaterial-based films. ACS Sustain. Chem. Eng..

[11] Pathak VM, Navneet (2017). Review on the current status of polymer degradation: A microbial approach. Bioresour. Bioprocess..

[12] Fadel SMM, Abou-Elseoud WSS, Hassan EAA, Ibrahim S, Hassan MLL (2022). Use of sugar beet cellulose nanofibers for paper coating. Ind. Crop Prod..

[13] Hassan EA, Hassan ML, Abou-Zeid RE, El-Wakil NA (2016). Novel nanofibrillated cellulose/chitosan nanoparticles nanocomposites films and their use for paper coating. Ind. Crop Prod..

[14] Huang S (2022). Cellulose nanofibers/polyvinyl alcohol blends as an efficient coating to improve the hydrophobic and oleophobic properties of paper. Sci. Rep..

[15] Ding Q (2019). Effect of nanocellulose fiber hornification on water fraction characteristics and hydroxyl accessibility during dehydration. Carbohydr. Polym..

[16] Kjellgren H, Gällstedt M, Engström G, Järnström L (2006). Barrier and surface properties of chitosan-coated greaseproof paper. Carbohydr. Polym..

[17] Park HJ (2000). Grease resistance and mechanical properties of isolated soy protein-coated paper. J. Am. Oil. Chem. Soc..

[18] Sirvio JA, Kolehmainen A, Liimatainen H, Niinimaki J, Hormi OEO (2014). Biocomposite cellulose-alginate films: Promising packaging materials. Food Chem..

[19] Du P (2021). The fluorine-free coating has excellent hydrophobic and oleophobic properties for porous cellulose-based materials. Cellulose.

[20] Xie JX (2020). Facile synthesis of fluorine-free cellulosic paper with excellent oil and grease resistance. Cellulose.

[21] Lee JY (2014). Fundamental study on developing wood powder as an additive of paperboard. Tappi J..

[22] Scurlock JM, Dayton DC, Hames B (2000). Bamboo: An overlooked biomass resource?. Biomass Bioenergy.

[23] Tayeb AH, Tajvidi M, Bousfield D (2020). Paper-based oil barrier packaging using lignin-containing cellulose nanofibrils. Molecules.

[24] Agarwal C, Csoka L (2018). Functionalization of wood/plant-based natural cellulose fibers with nanomaterials: A review. Tappi J..

[25] Kim H-J, Roy S, Rhim J-W (2021). Effects of various types of cellulose nanofibers on the physical properties of the CNF-based films. J. Environ. Chem. Eng..

[26] Tripathi S, Mehrotra G, Dutta P (2009). Physicochemical and bioactivity of cross-linked chitosan–PVA film for food packaging applications. Int. J. Biol. Macromol..

[27] Chio C, Sain M, Qin W (2019). Lignin utilization: A review of lignin depolymerization from various aspects. Renew. Sustain. Energy Rev..

[28] Wang W (2019). Lignin redistribution for enhancing barrier properties of cellulose-based materials. Polymers.

[29] Wang Z, Lin S (2017). The impact of low-surface-energy functional groups on oil fouling resistance in membrane distillation. J. Membr. Sci..

[30] Cai Q (2018). Dissolving process of bamboo powder analyzed by FT-IR spectroscopy. J. Mol. Struct..

[31] Hundhausen U, Militz H, Mai C (2009). Use of alkyl ketene dimer (AKD) for surface modification of particleboard chips. Eur. J. Wood Wood Prod..

[32] Li L, Neivandt DJ (2019). The mechanism of alkyl ketene dimer (AKD) sizing on cellulose model films studied by sum frequency generation vibrational spectroscopy. Cellulose.

[33] Segal L, Creely JJ, Martin AE, Conrad CM (1959). An empirical method for estimating the degree of crystallinity of native cellulose using the X-ray diffractometer. Text. Res. J..

[34] Liu YF (2014). A novel approach for the preparation of nanocrystalline cellulose by using phosphotungstic acid. Carbohydr. Polym..

[35] Abe K, Yano H (2009). Comparison of the characteristics of cellulose microfibril aggregates of wood, rice straw and potato tuber. Cellulose.

[36] de Souza Lima MM, Borsali R (2004). Rodlike cellulose microcrystals: Structure, properties, and applications. Macromol. Rapid Commun..

[37] Jang J-H (2020). Changes in the dimensions of lignocellulose nanofibrils with different lignin contents by enzymatic hydrolysis. Polymers.

[38] Li XL, Sun CJ, Zhou BX, He Y (2015). Determination of hemicellulose, cellulose and lignin in moso bamboo by near infrared spectroscopy. Sci. Rep..

[39] Wu K (2018). Fenton reaction-oxidized bamboo lignin surface and structural modification to reduce nonproductive cellulase binding and improve enzyme digestion of cellulose. ACS Sustain. Chem. Eng..

[40] Svagan AJ, Hedenqvist MS, Berglund L (2009). Reduced water vapour sorption in cellulose nanocomposites with starch matrix. Compos. Sci. Technol..

[41] Vu T, Chaffee A, Yarovsky I (2002). Investigation of lignin-water interactions by molecular simulation. Mol. Simul..

[42] Park H, Park SY, Yook S, Kim T-Y, Youn HJ (2020). Impregnation of paper with cellulose nanofibrils and polyvinyl alcohol to enhance durability. Nord Pulp Pap. Res. J..

[43] Atifi S, Miao C, Hamad WY (2017). Surface modification of lignin for applications in polypropylene blends. J. Appl. Polym. Sci..

[44] Zhaoyang Y, Yangbing W (2018). Enhancement of hydrophobicity of nanofibrillated cellulose through grafting of alkyl ketene dimer. Cellulose.

[45] Bildik AE, Hubbe MA, Gurboy KB (2016). Alkyl ketene dimer (AKD) sizing of paper under simplified treatment conditions. Tappi J..

